# Higher Dietary Acid Load Might Be a Potent Derivative Factor for Multiple Sclerosis: The Results from a Case–Control Study

**DOI:** 10.3390/nu15153311

**Published:** 2023-07-26

**Authors:** Zahra Saeedirad, Shadi Ariyanfar, Morvarid Noormohammadi, Zeinab Ghorbani, Abdorreza Naser Moghadasi, Sahar Shahemi, Milad Ghanaatgar, Nasim Rezaeimanesh, Azita Hekmatdoost, Amir Ghaemi, Soodeh Razeghi Jahromi

**Affiliations:** 1 Department of Clinical Nutrition and Dietetics, Faculty of Nutrition and Food Technology, Shahid Beheshti University of Medical Sciences, Tehran 19816-19573, Iran; maryamsaeedirad73@gmail.com (Z.S.); sahar_shahemi@yahoo.com (S.S.); miladghanaatgar@yahoo.com (M.G.); rezaeimaneshnasim@gmail.com (N.R.); a_hekmat2000@yahoo.com (A.H.); 2 Department of Human Nutrition, Foods, and Exercise, College of Agriculture and Life Science, Virginia Tech, Blacksburg, VA 24060, USA; ariyanfar@vt.edu; 3 Department of Nutrition, School of Public Health, Iran University of Medical Sciences, Tehran 14496-14535, Iran; 4 Cardiovascular Diseases Research Center, Department of Cardiology, Heshmat Hospital, School of Medicine, Guilan University of Medical Sciences, Rasht 41937-1311, Iran; dr.zeinab.ghorbani@gmail.com; 5 Department of Clinical Nutrition, School of Medicine, Guilan University of Medical Sciences, Rasht 41937-1311, Iran; 6 Multiple Sclerosis Research Center, Neuroscience Institute, Tehran University of Medical Sciences, Tehran 14167-53955, Iran; abdorrezamoghadasi@gmail.com; 7 Nutrition and Endocrine Research Center, Research Institute for Endocrine Sciences, Shahid Beheshti University of Medical Sciences, Tehran 19839-69411, Iran; 8 Department of Virology, Pasteur Institute of Iran, Tehran 13169-43551, Iran; ghaem_amir@yahoo.com

**Keywords:** multiple sclerosis, dietary acid load, potential renal acid load, net endogenous acid production, plant-based protein, animal-based protein, case–control study

## Abstract

This study aimed to investigate the association between dietary acid load (DAL) and multiple sclerosis (MS), through the potential renal acid load (PRAL) and net endogenous acid production (NEAP) scores. In a hospital-based case–control study of 109 patients with MS and 130 healthy individuals, a validated 168-item semi-quantitative food frequency questionnaire and a logistic regression model were used to evaluate the association between the DAL and MS. After adjusting for age (years), gender (male/female), body mass index (Kg/m^2^), and total calories (Kcal), the MS odds were 92% lower for those in the highest tertile of total plant-based protein (OR: 0.08, 95%CI: 0.03, 0.23; *p*-value < 0.001) and about four times higher for those in the highest tertile of the PRAL (OR: 4.16, 95%CI: 1.94, 8.91; *p*-value < 0.001) and NEAP scores (OR: 3.57, 95%CI: 1.69, 7.53; *p*-value < 0.001), compared to those in the lowest tertile. After further adjusting for sodium, saturated fatty acid, and fiber intake, the results remained significant for total plant-based protein intake (OR: 0.07, 95%CI: 0.01, 0.38; *p*-value = 0.002). In conclusion, a higher NEAP or PRAL score may be associated with increased odds of MS, while a higher intake of plant-based protein instead of animal-based protein may be protective.

## 1. Introduction

Multiple sclerosis (MS) is an inflammatory autoimmune disease of the central nervous system (CNS), with an unknown cause [1]. MS typically manifests between the ages of 20 and 40, and is the second-leading cause of non-traumatic disability among young adults. It is estimated that in 2022, about 2.8 million people worldwide had MS [2,3]. 

Inflammation is a common feature throughout all the stages of MS, with the acute phases exhibiting more apparent inflammation than the chronic phases. At the onset of the disease, peripheral immune cells, such as macrophages and CD8+ T cells, invade the CNS, due to disruption of the blood–brain barrier. As the disease progresses, the relative proportion of invading B cells and plasma cells increases. Over time, the inflammation within the CNS becomes more structured, and fewer invading cells are detected in lesions as the disease progresses. The formation of tertiary lymphoid structures in the meninges has been observed in secondary progressive MS. These inflammatory aggregates may cause additional demyelination and tissue damage in the later stages of the disease [4].

The typical lifespan of a person with MS is slightly reduced, but many people with relapsing–remitting MS (RRMS) live for decades without persistent disability. However, about half of RRMS patients will progress to secondary progressive MS within 10 years of the disease onset [5], which usually causes more severe disability. As a result, an average MS patient with disability can expect to live for about 40 years or more with the condition. A growing body of evidence suggests that diet is a modifiable factor that can influence MS risk, in addition to social, environmental, and psychological factors.

Several retrospective studies have reported an inverse relationship between MS risk and healthy dietary patterns, such as the vegetarian-based [6] and Mediterranean diets [7,8]. For example, a high-animal-fat dietary pattern has been associated with a higher risk of MS, while vegetarian and lacto-vegetarian dietary patterns have been associated with a lower risk [6]. A higher adherence to a Mediterranean diet through an increased intake of fruit and vegetables has been inversely associated with MS risk [7]. Similarly, a recent study found that adherence to a Mediterranean diet was protective against MS, compared to a Western dietary pattern [8]. The biological pathways through which these dietary patterns influence the MS risk involve changes in inflammation due to changes in the omega-3/omega-6 polyunsaturated fatty acid (PUFA) ratios, the regulation of the gut microbiota function, and the modification of mitochondrial bioenergetics [9]. These dietary patterns typically include foods with a high alkaline content, such as fruit and vegetables, and restrict the consumption of protein- and phosphorus-rich animal-based foods. The endogenous synthesis of the acid and base is primarily governed by the consumption of foods high in sulfur and with a high phosphorus content (potential acid precursors), and those with a high alkaline content, respectively [9,10].

The dietary acid load can have a significant impact on the functioning of the immune system. High acid levels can lead to a decreased white blood cell function, increased inflammation, reduced antibody production, and impaired T-cell function [10]. Additionally, a high dietary acid load can lead to changes in the microbiome composition, which can further impair immune system health [11]. Indicators of dietary acid load include the potential renal acid load (PRAL) and net endogenous acid production (NEAP), as well as the ratio of animal protein to potassium (A:P) [12]. The PRAL metric measures the alkalinity or acidity induced in the bloodstream by the dietary intake, based on the release of base or acid precursors after metabolism. The PRAL metric is based on five nutrients: protein, phosphorus, potassium, magnesium, and calcium. The NEAP metric employs two nutrients, protein and potassium, which have been shown to be indicative of dietary-induced acidity or alkalinity [12,13]. To our knowledge, no study had previously investigated the correlation between dietary acid load and MS risk. Therefore, the present study aimed to investigate the association between dietary acid load and MS risk, using the PRAL and NEAP scores.

## 2. Materials and Methods

### 2.1. Ethical Considerations

The Institutional Review Board of the National Institute for Medical Research Development (NIMAD) (Research number = 962667), the research ethics committee of NIMAD, and the research ethics board of the Sina MS research center at Tehran University of Medical Sciences (Ethics code: IR.NIMAD.REC.1396.320) approved the study’s protocol. The research adhered to the Helsinki Declaration of 1964 and its subsequent revisions. Each participant provided written consent to participate in the research.

### 2.2. Study Participants

This hospital-based case–control study was conducted in Tehran, Iran, between January 2018 and January 2021. The sampling and methods are detailed in our previous study [14]. Briefly, MS patients, who had been diagnosed between one week and one month prior, were selected from a referral university hospital MS clinic, using a convenience-sampling approach. The MS diagnosis was carried out by a neurologist and MS specialist using a combination of a physical examination, the patient history, magnetic resonance imaging (MRI), and the revised McDonald diagnostic criteria [15]. The drugs used by the patients were in the category of monoclonal antibodies (rituximab, natalizumab, ocrelizumab), immunomodulators (glatiramer acetate, interferon beta-1a, interferon beta-1b, teriflunomide), Nrf2 activators (dimethyl fumarate), sphingosine 1-phosphate receptor modulators (fingolimod), and immunosuppressants (azathioprine). The severity of the disease was similar among the MS patients. Healthy controls were selected from the hospital’s emergency department. All participants were between 18 and 50 years old. Individuals who followed a specific diet in the preceding year, consumed dietary supplements, were pregnant or lactating, had other variants of MS, received corticosteroid injections (pulse therapy), had experienced an RRMS relapse within the month prior to sampling, had neurological disorders other than MS, or had chronic illnesses or metabolic disorders that affected their dietary intake (e.g., diabetes or chronic kidney disease) were excluded from the study. The control group participants were not taking any medication. Samples were also excluded if the participant had a body mass index (BMI) lower than 18.5 or higher than 34.9 kg/m^2^, left more than 70 items blank on the food frequency questionnaire (FFQ), or reported an energy intake lower than 800 Kcal or higher than 4200 Kcal.

### 2.3. Demographic and Anthropometric Data

The demographic and anthropometric data measurements are provided in detail [14]. Data concerning age, adherence to a specific diet, and drug use history were collected from all participants by a verified nutrition specialist during a personal interview. A Seca digital body weight scale with an accuracy of 100 g, and a tape measure with an accuracy of 1 cm were used to collect the data on weight and height, respectively. The measurements were taken while the participants were standing in light clothing and without shoes. The BMI was calculated by dividing the weight in kilograms by the square of the height in meters.

### 2.4. Dietary Assessments

A verified nutrition specialist conducted a personal interview with the participants to gather data on their usual diet over the preceding year. This was done using a Willett-format semi-quantitative FFQ, consisting of 168 food items. The reliability and validity of the questionnaire have been reported for the Iranian population in studies [16,17,18,19]. The mean portion size of food items was shown to the participants, and they reported the frequency of their intake of the food items (daily, weekly, monthly, or yearly). Subsequently, the weight of each food item was transformed into grams using the information presented in the household scale guide. The total calorie and micro/macronutrient intake of each participant were computed, using the Iranian food composition table [20], and the food composition data of the United States Department of Agriculture [21].

### 2.5. Dietary Acid Load

The three distinct methodologies for measuring the dietary acid load are referred to as the PRAL, NEAP, and protein/potassium ratio. All have been validated in previous studies [12,22] and are presented in detail in our previous study [23], as follows: (1) PRAL (mEq/day) = (0.49 × protein (g/day)) + (0.037 × phosphorus (mg/day)) − (0.021 × potassium (mg/day)) − (0.026 × magnesium (mg/day)) − (0.013 × calcium (mg/day)) [12]; (2) NEAP (mEq/day) = (54.5 × protein intake (g/day0 ÷ potassium intake (mEq/day)) − 10.2 [13]; and (3) protein/potassium ratio = total protein/potassium intake (both g/day) [22].

### 2.6. Statistical Analysis

The data analysis was carried out using SPSS software (v.26). The normal distribution of the quantitative variables was assessed separately in each group, using skewness, histogram charts, Q–Q plots, stem-and-leaf plots, box plots, and the Kolmogorov–Smirnov test. The independent two-sample t-test was used to compare the quantitative data between the case and control groups. The Mann–Whitney U test was employed to examine data that were not normally distributed. The chi-squared test was used to compare the qualitative variables between the cases and controls. To explore the relationship between MS and the dietary acid load components, the variables were split into tertiles based on the control intakes. The odds ratio (OR) with a 95% CI for each tertile was calculated using logistic regression models, with the first tertile serving as the reference group. The basic binary logistic regression models contained age and gender as variables. To address potential confounding factors, binary logistic regression model 1 was employed, incorporating the BMI (kg/m^2^) and total calories (Kcal). Furthermore, model 2 was adjusted for the intake of sodium (mg/day), saturated fatty acids (gr/day), and fiber (gr/day). Statistical significance was attributed to *p*-values less than 0.05.

## 3. Results

In the study, 109 MS patients (84 females) and 130 healthy controls (92 females) were included. The median (Q1–Q3) age in the control group was significantly higher than that of the case group (35 (29, 42) and 33 (29, 38), respectively; *p*-value = 0.040). Additionally, the mean ± SD of the BMI in the control group was significantly higher than in the MS patients (26.92 ± 3.52 and 25.18 ± 3.86, respectively; *p*-value < 0.001). The total intake of calories and macro/micronutrients in the patients with MS and the healthy controls are represented in Table 1. Furthermore, there was no difference between sexes according to the BMI (*p*-value = 0.645), PRAL (*p*-value = 0.827), and NEAP (*p*-value = 0.483) scores (Supplementary Table S1).

The total intake of calories and micro/macronutrients according to the PRAL and NEAP tertiles is provided in Table 2 and Table 3.

In the base model adjusted for age (years) and gender (male/female), the odds of MS were 2.28 times higher for those in the last tertile of total animal-based protein (OR: 2.28, 95%CI: 1.18, 4.41; *p*-value = 0.013), and about five times higher in the last tertile of the PRAL score (OR: 4.88, 95%CI: 2.38, 10.02; *p*-value < 0.001) and NEAP score model (OR: 5.03, 95%CI: 2.47, 10.26; *p*-value < 0.001), compared to the first tertile. In model 1, additionally adjusted for BMI (Kg/m2) and total calories (Kcal), the MS odds were 92% lower for those in the last tertile of total plant-based protein (OR: 0.08, 95%CI: 0.03, 0.23; *p*-value < 0.001), and about four times higher for those in the last tertile of the PRAL (OR: 4.16, 95%CI: 1.94, 8.91; *p*-value < 0.001) and NEAP score (OR: 3.57, 95%CI: 1.69, 7.53; *p*-value < 0.001) than in the first tertile. In model 2, additionally adjusted for sodium (mg/day), saturated fatty acids (gr/day), and fiber (gr/day) intake, the patients in the last tertile of total plant-based protein had 93% lower odds of MS (OR: 0.07, 95%CI: 0.01, 0.38; *p*-value = 0.002). A significant direct association was observed between protein/potassium ratio and odds of MS in both the base (OR: 5.03, 95%CI: 2.47, 10.26; *p*-value < 0.001) and the first model (OR: 3.57, 95%CI: 1.69, 7.53; *p*-value < 0.001) (Table 4).

## 4. Discussion

In the current study, after adjusting for age, gender, BMI, and total calories, a higher dietary acid load, defined by a higher NEAP or PRAL score, was correlated with increased odds of MS. Moreover, the MS odds were increased in individuals with a higher protein/potassium ratio. In addition, a higher total plant-based protein intake was associated with reduced odds of MS, which was the only variable that remained statistically significant after further adjustment for sodium, saturated fatty acids, and fiber intake. Food items rich in sodium and saturated fatty acids, compared to those rich in fiber, are shown to have a higher dietary acid load, so their adjustment in the last model may have affected the results observed in the previous models.

Foods that increase a diet’s acidity levels include meat, fish, grains, and cheese, while foods that increase a diet’s alkalinity levels include fruit, vegetables, milk, and yogurt [11]. With regard to acid- and base-inducing foods, the affirmative association between MS risk and dietary acid load supports prior studies on dietary intake and MS risk. In a study on RRMS patients, a higher consumption of meat products was directly associated with the odds of RRMS. In contrast, dietary patterns rich in whole grains, vegetables, legumes, nuts, and fruit showed protective effects against RRMS [6]. Moreover, evidence showed that the Mediterranean diet had an anti-inflammatory effect, and reduced fatigue, in MS patients [24]. In another study on MS patients, a diet consisting of whole grains, vegetables, fruit, legumes, and limited added sugar and red meat was related to reduced disability and depression among MS patients [25]. A higher consumption of refined carbohydrates also showed a direct association with a higher MS risk, but fruit and vegetable intake might decrease the risk of MS [7]. As demonstrated in our previous study, the MIND diet and its constituents, such as green leafy vegetables, other vegetables, and beans, appear to lower the likelihood of MS, whereas pastries and sweets, cheese, poultry, and fried/fast foods had a contrary impact [14].

Higher levels of dietary acid load are associated with an increased insulin resistance [26], and reducing the dietary acid load may be beneficial in improving glucose tolerance, and potentially reducing the risk of insulin-resistance-related health conditions [27]. On the other hand, there is a strong association between insulin resistance and MS [28]. Studies have found that individuals with MS are more likely to have insulin resistance than those without MS, and that higher levels of insulin resistance are associated with greater disability in MS patients [29]. Insulin resistance is thought to be due to the presence of pro-inflammatory cytokines in the body, which are known to be elevated in people with MS [30].

An acidic diet can lead to an imbalance in the body’s pH levels, which can lead to a decrease in white blood cell production. Moreover, certain specific foods can further decrease white blood cell function, such as processed foods and meat, which can reduce the number of circulating white blood cells, due to their high acidity [31]. A decrease in white blood cell function has been observed in multiple sclerosis [32].

An increase in the dietary acid load can trigger an autoreactive T-cell response [33]. Usually, a highly acid-inducing diet is poor in fiber and high in animal-based proteins. A diet poor in fiber could result in a sharp increase in the serum glucose levels, followed by a rise in insulin secretion, which upregulates the production of arachidonic acid, and enhances inflammation [34]. A high intake of animal-based protein leads to gut dysbiosis, which disrupts the immune system by developing T-regulatory (Treg) cells, which activate the inflammatory pathway by increasing cytokines such as Interleukin-6 [35]. Gut microbial dysbiosis is liable to cause inflammatory responses, which result in neuroinflammation and degeneration in the brain [36,37]. The gut microbiota metabolizes fiber to short-chain fatty acids, which play an immunomodulatory role by reducing pro-inflammatory cytokine production, and improving the intestinal integrity [35].

A higher dietary acid load increases the number of fat cells, and consequently leads to obesity [38]. A systematic review and meta-analysis suggested a positive correlation between higher triglyceride concentrations and prevalence of obesity with a higher dietary acid load content, as evidenced by high PRAL scores. In the subgroup analysis, an association was found between higher PRAL scores and higher BMIs in women. Reducing the content of the dietary acid load may prove to be a useful strategy for preventing obesity and metabolic disorders [38]. Childhood and/or early adulthood obesity has been reported to increase the risk of MS [39]. Obesity can also worsen the course of the disease [40]. One of the proposed mechanisms is the pro-inflammatory state caused by obesity. Moreover, IGF-1 and insulin, which frequently have high serum levels in overweight/obese individuals, promote the PI3K/Akt/mTOR activation that subsequently leads to inflammation [41]. Additionally, after each meal, one might experience transient postprandial inflammation, depending on the number of calories, as well as on the quantity and type of food [34].

Moreover, an investigation into the comorbidities of MS has shown that individuals with MS have a lower bone mineral density, and a greater incidence of osteoporosis than those of a similar age and gender who do not have MS. This may contribute to the disability experienced by these patients [42]. According to studies, there is an association between the dietary acid load and a reduced bone mineral density [43]. As shown by a systematic review and meta-analysis, there is a substantial negative correlation between the NEAP and bone mineral density [44]. Furthermore, depression is a common comorbidity in patients with MS [45]. Various studies in Iran and globally have examined the relationship between dietary patterns and the incidence of depression, and have shown that following dietary patterns similar to the Western dietary pattern, rich in animal products that have a high dietary acid load, compared to plant-based foods that have a lower dietary acid load, is associated with a higher incidence of depression [46,47]. Moreover, a substantial positive correlation exists between the dietary acid load and the likelihood of depression [48]. Therefore, it seems that following a dietary pattern with a lower dietary acid load is effective in reducing MS comorbidities.

The present study had several strengths. We used a validated FFQ, and individuals with an energy intake lower than 800 or higher than 4200 Kcal were excluded. In order to attain a causal interpretation, and diminish recall bias, newly diagnosed MS patients were included. The participation rates were high in both individuals with MS, and healthy controls. The dietician who specialized in the field was blinded to the diagnostic results while conducting the interview using questionnaires. Nevertheless, we encountered certain limitations pertaining to measurement bias and recall bias. The cultural and religious prohibition of alcohol and opium in Iran hindered our ability to gather data on these variables. Moreover, the inflammatory biomarkers associated with the examined serum samples were not analyzed, along with the serum and urinary pH.

## 5. Conclusions

After accounting for various factors, the study found that a higher dietary acid load, as indicated by a higher NEAP or PRAL score, was associated with an increased likelihood of MS. Additionally, individuals with a higher protein/potassium ratio had an increased likelihood of MS. On the other hand, a higher intake of plant-based protein was linked to a reduced likelihood of MS, even after further adjustments for sodium, saturated fatty acids, and fiber intake. Further studies with other designs, such as cohorts and clinical trials, are needed in order to clarify the effects observed in this study.

## Figures and Tables

**Table 1:** The demographic characteristics and total intake of calories and macro/micronutrients in the patients with multiple sclerosis and in the healthy controls ^1^.

Variables ^2^	Healthy Controls (*n* = 130)	Patients with Multiple Sclerosis (*n* = 109)	*p*-Value
Age (years)	35 (29, 42)	33 (29, 38)	0.040
Sex, female, frequency (percentages)	92 (70.8)	84 (77.1)	0.271
Body mass index, Kg/m^2^, Mean (SD)	26.92 ± 3.52	25.18 ± 3.86	<0.001
Total calorie intake (Kcal/day)	2303.04 (1857.29, 2663.53)	2542.49 (2143.03, 3109.57)	<0.001
Protein (gr/day)	82.14 ± 25.33	85.65 ± 26.59	0.298
Carbohydrates (gr/day)	289.49 (225.25, 350.44)	331.06 (281.07, 426.16)	<0.001
Fat (gr/day)	88.53 (72.64, 112.04)	100.07 (83.44, 128.11)	0.002
Cholesterol (mg/day)	270.78 (202.89, 377.31)	319.59 (243.18, 397.73)	0.055
Saturated fatty acids (gr/day)	30.08 (23.63, 36.64)	35.89 (27.12, 48.39)	<0.001
Sodium (mg/day)	3468.27 (2724.48, 4414.04)	7764.76 (5058.46, 9057.54)	<0.001
Fiber (gr/day)	23.18 (19.10, 31.23)	19.61 (15.80, 26.79)	0.001
Trans fatty acids (gr/day)	2.31 (1.45, 7.68)	2.94 (1.93, 5.23)	0.555
Poly unsaturated fatty acids (gr/day)	28.38 (22.13, 35.71)	27.55 (22.62, 32.83)	0.227
Iron (mg/day)	17.02 (13.35, 22.20)	15.71 (12.86, 19.13)	0.053
Calcium (mg/day)	1002.45 ± 380.51	952.22 ± 330.81	0.282
Folate (mcg/day)	416.04 (327.31, 531.16)	300.90 (242.53, 361.99)	<0.001
Sugar (gr/day)	83.06 (65.56, 110.00)	105.98 (80.30, 140.59)	<0.001
Glucose (gr/day)	10.95 (7.91, 16.16)	19.02 (12.35, 29.14)	<0.001
Vitamin A (RAE/day)	1287.93 (915.67, 2212.71)	879.30 (625.63, 1301.77)	<0.001
Vitamin D (mcg/day)	1.12 (0.40, 2.27)	1.05 (0.40, 1.63)	0.346
Vitamin C (mg/day)	141.89 (97.06, 202.01)	117.90 (88.96, 162.63)	0.034
Vitamin E (mg/day)	3.92 (3.02, 5.60)	5.02 (3.88, 6.21)	<0.001
Beta carotene (mg/day)	649.17 (368.51, 1325.09)	250.40 (127.84, 403.39)	<0.001
Mono unsaturated fatty acids (gr/day)	32.84 (26.11, 40.35)	39.48 (31.24, 49.20)	<0.001
Phosphorus (mg/day)	1201.42 (912.80, 1567.49)	1101.12 (901.42, 1362.15)	0.028
Magnesium (mg/day)	298.85 (233.58, 376.01)	233.17 (198.20, 274.09)	<0.001
Potassium (mg/day)	3770.32 (2956.91, 4581.18)	2976.51 (2500.05, 3593.60)	<0.001
Zinc (mg/day)	9.99 (7.68, 12.17)	9.82(7.90, 11.92)	0.885
Copper (mg/day)	1.63 (1.13, 2.36)	1.38 (1.08, 1.81)	0.025
Manganese (mg/day)	3.52 (2.84, 4.48)	2.66 (2.17, 3.43)	<0.001
Selenium (µmol/day)	0.58 (0.34, 1.09)	1.12 (0.63, 1.19)	<0.001
Vitamin B1 (mg/day)	10.29 (8.14, 13.25)	8.74 (6.91, 11.01)	<0.001
Vitamin B2 (mg/day)	1.72 (1.31, 2.18)	1.54 (1.27, 1.82)	0.015
Vitamin B3 (mg/day)	1.61 (1.09, 2.06)	1.46 (1.12, 1.81)	0.111
Vitamin B6 (mg/day)	18.82 (14.03, 23.65)	20.65 (16.73, 25.70)	0.016
Vitamin B12 (mcg/day)	1.32 (1.03, 1.79)	1.19 (1.00, 1.52)	0.027
Vitamin B5 (mg/day)	5.28 (3.42, 7.61)	5.81 (3.87, 9.03)	0.152
Vitamin B8 (mcg/day)	5.50 (4.13, 6.94)	4.82 (4.10, 5.60)	0.004
Vitamin K (mcg/day)	21.05 (16.53, 28.10)	20.01 (16.28, 25.93)	0.271
Caffeine (mg/day)	173.64 (101.00, 252.93)	55.71 (42.25, 82.16)	<0.001
PRAL	−13.62 (−25.37, −4.60)	−0.01 (−14.92, 9.29)	<0.001
NEAP	35.59 (30.23, 42.73)	48.09 (37.16, 63.15)	<0.001
Protein/potassium	0.021 (0.019, 0.024)	0.027 (0.022, 0.034)	<0.001

^1^ Using the Mann–Whitney U, *X*^2^ or independent samples t-test, as appropriate. ^2^ The values are median (Q1–Q3) unless otherwise noted. BMI, body mass index; PRAL, potential renal acid load; NEAP, net endogenous acid production.

**Table 2:** The demographic characteristics and total intake of calories and macro/micronutrients in the patients with multiple sclerosis and in the healthy controls, according to the tertiles of the PRAL ^1^.

Tertiles of PRAL	T1 (*n* = 57; Cases = 14)	T2 (*n* = 68; Cases = 24)	T3 (*n* = 114; Cases = 71)	*p*-Value
Age (years)	35 (29.5, 42)	35 (29, 43.75)	32 (29, 39)	0.102
Body mass index	27.83 (24.79, 30.49)	26.52 (23.03, 29.61)	24.68 (22.84, 27.36)	<0.001
Total calorie intake (Kcal/day)	2455.61 (1923.21, 2764.37)	2179.55 (1787.31, 2649.86)	2489.45 (2115.30, 3064.67)	0.009
Protein (gr/day)	84.48 (65.51, 100.43)	71.81 (57.18, 89.03)	90.69 (72.08, 106.32)	<0.001
Carbohydrates (gr/day)	334.05 (234.74, 413.91)	298.32 (232.52, 361.86)	317.73 (256.07, 395.17)	0.283
Fat (gr/day)	90.87 (74.19, 108.71)	88.45 (71.73, 106.73)	103.95 (81.39, 126.31)	0.002
Cholesterol (mg/day)	253.75 (195.76, 361.65)	270.84 (196.16, 354.20)	322.65 (249.41, 425.16)	0.002
Saturated fatty acids (gr/day)	29.27 (23.45, 34.97)	28.92 (22.72, 36.70)	36.67 (27.43, 44.75)	<0.001
Sodium (mg/day)	4404.13 (3018.33, 6912.04)	4291.07 (3387.02, 7211.30)	5187.42 (3361.90, 8632.02)	0.126
Fiber (gr/day)	31.44 (25.64, 38.09)	21.71 (18.87, 26.45)	18.70 (15.03, 23.16)	<0.001
Trans fatty acids (gr/day)	2.05 (1.33, 5.49)	2.31 (1.34, 6.14)	3.36 (1.96, 6.82)	0.026
Poly unsaturated fatty acids (gr/day)	29.61 (22.26, 36.67)	28.51 (20.49, 33.25)	27.77 (23.34, 33.67)	0.517
Iron (mg/day)	19.08 (13.89, 23.93)	14.96 (12.22, 18.86)	16.00 (12.98, 19.98)	0.060
Calcium (mg/day)	1129.71 (932.33, 1386.36)	854.27 (714.04, 1032.45)	956.18 (712.14, 1165.62)	<0.001
Folate (mcg/day)	448.29 (376.26, 595.81)	353.07 (294.81, 411.45)	305.94 (234.99, 385.30)	<0.001
Sugar (gr/day)	110.89 (85.48, 143.11)	83.61 (69.34, 122.10)	91.28 (67.52, 111.28)	0.003
Glucose (gr/day)	16.13 (11.82, 25.24)	12.21 (8.72, 20.43)	14.41 (9.27, 23.50)	0.085
Vitamin A (RAE/day)	1691.52 (1132.61, 2498.88)	1039.54 (745.08, 1617.16)	943.72 (600.93, 1334.02)	<0.001
Vitamin D (mcg/day)	1.65 (0.67, 2.40)	0.75 (0.36, 1.36)	0.95 (0.40, 1.71)	0.001
Vitamin C (mg/day)	214.97 (169.30, 263.73)	144.07 (109.42, 176.59)	101.28 (74.09, 130.19)	<0.001
Vitamin E (mg/day)	5.10 (3.62, 6.43)	3.83 (3.06, 5.68)	4.51 (3.65, 5.59)	0.008
Beta carotene (mg/day)	967.67 (451.67, 1637.04)	466.47 (308.46, 726.69)	270.82 (130.35, 497.51)	<0.001
Mono unsaturated fatty acids (gr/day)	34.77 (26.96, 41.78)	35.60 (25.38, 41.59)	38.38 (30.06, 47.03)	0.019
Phosphorus (mg/day)	1397.21 (1024.42, 1646.48)	981.50 (846.87, 1215.95)	1161.92 (897.13, 1460.60)	<0.001
Magnesium (mg/day)	358.06 (291.29, 434.20)	248.59 (206.39, 298.06)	234.08 (192.43, 296.33)	<0.001
Potassium (mg/day)	4828.35 (3851.84, 5584.11)	3199.99 (2780.45, 3915.69)	2922.91 (2371.45, 3606.47)	<0.001
Zinc (mg/day)	10.62 (8.70, 12.44)	8.44 (7.14, 10.37)	10.68 (8.05, 12.85)	0.001
Copper (mg/day)	1.78 (1.35, 2.67)	1.30 (1.03, 1.80)	1.42 (1.08, 1.96)	<0.001
Manganese (mg/day)	3.95 (3.12, 4.92)	3.13 (2.40, 3.64)	2.91 (2.13, 3.56)	<0.001
Selenium (µmol/day)	1.08 (0.37, 1.14)	0.67 (0.36, 1.14)	0.69 (0.49, 1.14)	0.664
Vitamin B1 (mg/day)	1.74 (1.38, 2.21)	1.53 (1.23, 1.90)	1.59 (1.29, 2.02)	0.084
Vitamin B2 (mg/day)	1.83 (1.33, 2.20)	1.28 (1.03, 1.69)	1.53 (1.15, 1.98)	0.001
Vitamin B3 (mg/day)	18.71 (14.54, 23.49)	17.55 (13.43, 22.75)	21.39 (17.31, 26.12)	<0.001
Vitamin B6 (mg/day)	1.63 (1.29, 1.99)	1.20 (1.00, 1.62)	1.15 (0.94, 1.41)	<0.001
Vitamin B12 (mcg/day)	4.63 (2.90, 7.16)	4.11 (3.20, 6.00)	6.26 (4.67, 9.40)	<0.001
Vitamin B5 (mg/day)	6.25 (4.99, 7.21)	4.59 (3.87, 5.77)	5.06 (4.09, 5.97)	<0.001
Vitamin B8 (mcg/day)	24.56 (19.77, 33.70)	18.08 (15.12, 23.65)	20.64 (16.46, 25.43)	<0.001
Vitamin K (mcg/day)	233.58 (114.81, 315.09)	118.22 (71.45, 166.93)	60.84 (44.64, 94.09)	<0.001
Caffeine (mg/day)	148.11 (98.80, 251.17)	126.20 (82.35, 199.95)	105.22 (62.22, 160.64)	0.011

^1^ Using the Kruskal–Wallis test; PRAL, potential renal acid load; T, tertile.

**Table 3:** The demographic characteristics and total intake of calories and macro/micronutrients in the patients with multiple sclerosis and in the healthy controls, according to the tertiles of the NEAP ^1^.

Tertiles of NEAP	T1 (*n* = 58; Cases = 15)	T2 (*n* = 64; Cases = 20)	T3 (*n* = 117; Cases = 74)	*p*-Value
Age (years)	35.5 (28, 42)	34.5 (29, 43.75)	32 (29, 38.5)	0.165
Body mass index	27.83 (25.37, 30.81)	25.55 (22.95, 29.19)	24.84 (23.03, 27.62)	0.001
Total calorie intake (Kcal/day)	2151.90 (1704.43, 2662.15)	2351.30 (1908.86, 2741.66)	2501.55 (2184.21, 3062.24)	0.001
Protein (gr/day)	70.66 (54.13, 89.85)	74.88 (58.50, 98.22)	90.82 (74.10, 106.63)	<0.001
Carbohydrates (gr/day)	290.18 (223.21, 367.43)	309.01 (249.65, 374.67)	324.74 (261.85, 402.44)	0.082
Fat (gr/day)	83.20 (67.58, 107.73)	86.68 (74.19, 113.26)	101.76 (86.07, 126.64)	<0.001
Cholesterol (mg/day)	235.58 (173.85, 319.09)	269.32 (205.38, 368.94)	333.99 (255.98, 425.46)	<0.001
Saturated fatty acids (gr/day)	27.42 (21.21, 33.39)	30.03 (23.73, 38.38)	35.76 (28.62, 44.97)	<0.001
Sodium (mg/day)	4293.05 (2913.74, 6711.94)	4238.50 (3424.67, 6948.42)	5639.32 (3575.15, 8714.47)	0.006
Fiber (gr/day)	28.38 (20.44, 35.21)	26.33 (20.99, 33.25)	28.20 (24.42, 33.89)	0.453
Trans fatty acids (gr/day)	14.75 (11.96, 20.49)	16.22 (12.81, 21.78)	16.78 (13.23, 20.88)	0.429
Poly unsaturated fatty acids (gr/day)	1019.86 (712.88, 1228.20)	920.18 (736.53, 1094.66)	970.13 (735.62, 1174.99)	0.603
Iron (mg/day)	413.44 (318.10, 569.96)	385.24 (297.55, 462.05)	318.03 (253.99, 387.57)	<0.001
Calcium (mg/day)	27.37 (21.69, 37.11)	22.68 (18.41, 31.73)	19.41 (15.62, 24.58)	<0.001
Folate (mcg/day)	2.21 (1.22, 7.64)	2.31 (1.44, 5.44)	3.30 (1.92, 6.43)	0.052
Sugar (gr/day)	105.89 (70.07, 135.07)	93.65 (71.05, 123.20)	90.94 (69.54, 115.28)	0.254
Glucose (gr/day)	14.33 (10.46, 22.88)	12.32 (8.84, 20.43)	14.46 (9.30, 23.61)	0.415
Vitamin A (RAE/day)	1368.27 (942.98, 2384.53)	1192.73 (745.08, 1751.19)	958.90 (668.98, 1342.54)	0.001
Vitamin D (mcg/day)	1.36 (0.22, 2.32)	0.96 (0.39, 1.59)	1.16 (0.47, 1.84)	0.668
Vitamin C (mg/day)	198.96 (135.44, 261.79)	152.12 (102.24, 193.02)	104.49 (79.05, 136.63)	<0.001
Vitamin E (mg/day)	4.00 (3.26, 6.35)	4.45 (3.63, 5.82)	4.52 (3.51, 5.60)	0.899
Beta carotene (mg/day)	794.79 (362.98, 1573.57)	486.78 (283.64, 822.95)	296.63 (131.24, 529.22)	<0.001
Mono unsaturated fatty acids (gr/day)	31.31 (25.92, 41.61)	35.90 (27.31, 40.17)	38.30 (30.27, 47.07)	0.009
Phosphorus (mg/day)	1195.65 (913.93, 1600.80)	1048.96 (831.67, 1408.12)	1168.35 (922.96, 1438.67)	0.494
Magnesium (mg/day)	298.61 (241.57, 427.25)	264.88 (212.73, 345.52)	244.25 (199.62, 296.85)	<0.001
Potassium (mg/day)	4012.29 (3151.91, 5448.55)	3469.59 (2691.21, 4463.24)	3000.35 (2460.01, 3616.19)	<0.001
Zinc (mg/day)	9.00 (7.36, 11.99)	9.47 (7.14, 11.96)	10.64 (8.40, 12.32)	0.072
Copper (mg/day)	1.52 (1.23, 2.53)	1.57 (1.07, 2.05)	1.41 (1.09, 1.94)	0.389
Manganese (mg/day)	3.48 (2.65, 4.92)	3.38 (2.62, 3.82)	2.91 (2.15, 3.60)	0.001
Selenium (µmol/day)	1.09 (0.38, 1.14)	0.65 (0.36, 1.13)	0.81 (0.50, 1.14)	0.478
Vitamin B1 (mg/day)	1.56 (1.24, 2.03)	1.67 (1.32, 2.04)	1.63 (1.31, 2.04)	0.590
Vitamin B2 (mg/day)	1.59 (1.03, 1.94)	1.35 (1.04, 1.92)	1.55 (1.18, 1.96)	0.401
Vitamin B3 (mg/day)	16.78 (12.39, 20.90)	18.08 (14.64, 23.43)	21.51 (18.06, 26.24)	<0.001
Vitamin B6 (mg/day)	1.51 (1.15, 1.91)	1.28 (0.95, 1.78)	1.19 (0.99, 1.43)	0.001
Vitamin B12 (mcg/day)	3.90 (2.53, 6.23)	4.29 (3.46, 8.01)	6.20 (4.71, 9.36)	<0.001
Vitamin B5 (mg/day)	5.58 (3.95, 6.86)	4.88 (4.14, 6.48)	5.13 (4.11, 5.98)	0.299
Vitamin B8 (mcg/day)	22.20 (16.11, 32.56)	20.69 (16.56, 27.49)	20.04 (16.53, 25.52)	0.518
Vitamin K (mcg/day)	186.04 (96.52, 292.34)	128.38 (70.81, 209.20)	62.76 (44.90, 103.02)	<0.001
Caffeine (mg/day)	116.13 (97.46, 253.81)	147.84 (99.31, 199.95)	106.41 (63.70, 170.85)	0.067

^1^ Using the Kruskal–Wallis test; NEAP, net endogenous acid production; T, tertile.

**Table 4:** The odds ratio and 95% confidence interval for multiple sclerosis, according to the tertiles of dietary acid load ^a^.

Indexes of Dietary Acid Load	Odds Ratio of Dietary Indexes of Acid Load (95% Confidence Interval)			
	1st	2nd	3rd	*p* for Trend
Total plant-based protein (gr/day)				
No. cases/no. controls	44/43	36/44	29/43	
Base model ^b^	1.00 (Ref.)	0.85 (0.45, 1.58)	0.73 (0.38, 1.40)	0.339
Model 1 ^c^	1.00 (Ref.)	0.29 (0.13, 0.63)	0.08 (0.03, 0.23)	<0.001
Model 2 ^d^	1.00 (Ref.)	0.29 (0.10, 0.90)	0.07 (0.01, 0.38)	0.002
Total animal-based protein (gr/day)				
No. cases/no. controls	24/43	34/44	51/43	
Base model ^b^	1.00 (Ref.)	1.47 (0.74, 2.93)	2.28 (1.18, 4.41)	0.013
Model 1 ^c^	1.00 (Ref.)	1.11 (0.53, 2.30)	0.87 (0.38, 2.01)	0.745
Model 2 ^d^	1.00 (Ref.)	1.41 (0.47, 4.22)	0.88 (0.24, 3.26)	0.833
PRAL (mEq/day)				
No. cases/no. controls	14/43	24/44	71/43	
Base model ^b^	1.00 (Ref.)	1.68 (0.76, 3.69)	4.88 (2.38, 10.02)	<0.001
Model 1 ^c^	1.00 (Ref.)	1.83 (0.79, 4.21)	4.16 (1.94, 8.91)	<0.001
Model 2 ^d^	1.00 (Ref.)	0.50 (0.13, 1.74)	0.86 (0.23, 3.18)	0.884
NEAP (mEq/day)				
No. cases/no. controls	15/43	20/44	74/43	
Base model ^b^	1.00 (Ref.)	1.31 (0.59, 2.91)	5.03 (2.47, 10.26)	<0.001
Model 1 ^c^	1.00 (Ref.)	1.01 (0.43, 2.36)	3.57 (1.69, 7.53)	<0.001
Model 2 ^d^	1.00 (Ref.)	0.41 (0.12, 1.46)	0.91 (0.25, 3.31)	0.800
Protein/potassium ratio				
No. cases/no. controls	15/43	20/44	74/43	
Base model ^b^	1.00 (Ref.)	1.31 (0.59, 2.91)	5.03 (2.47, 10.26)	<0.001
Model 1 ^c^	1.00 (Ref.)	1.01 (0.43, 2.36)	3.57 (1.69, 7.53)	<0.001
Model 2 ^d^	1.00 (Ref.)	0.41 (0.12, 1.46)	0.91 (0.25, 3.31)	0.800

^a^ Logistic regression model. ^b^ Adjusted for age (years), gender (male/female). ^c^ Additionally adjusted for BMI (Kg/m2) and total calories (Kcal). ^d^ Additionally adjusted for sodium (mg/day), saturated fatty acids, and fiber intake (gr/day). PRAL, potential renal acid load; NEAP, net endogenous acid production.

## Data Availability

The datasets used and/or analyzed during the current study are available from the corresponding author upon reasonable request.

## Supplementary Materials

The following supporting information can be downloaded at: https://www.mdpi.com/article/10.3390/nu15153311/s1, Table S1: Demographic characteristics and total intake of calories and macronutrient and dietary acid load in female and male.
