# Supplementary material for: Higher Dietary Acid Load Might Be a Potent Derivative Factor for Multiple Sclerosis: The Results from a Case–Control Study
Source: Nutrients. 2023 Jul 26;15(15):3311. doi: 10.3390/nu15153311 (PMC10420939; doi:10.3390/nu15153311)
Supplement: Supplementary file 1 [file nutrients-15-03311-s001.zip › nutrients-2467098-supplementary.pdf]

Supplementary Table 1. Demographic characteristics and total intake of calories and macronutrient and dietary acid load in female and male[1]

Variables[2]	Female (n = 176)	Male (n = 63)	P-value
Age (years)	34 (29, 41)	34 (30, 42)	0.661
Body mass index, Kg/m^2, Mean (SD)	26.19 ± 3.98	25.96 ± 3.15	0.645
Total calories intake (Kcal/day)	2319.52 (1906.24, 2754.85)	2556.18 (2272.29, 3060.90)	0.002
Protein (gr/day)	78.95 (60.13, 98.86)	89.22 (74.25, 106.83)	0.008
Carbohydrates (gr/day)	298.71 (235.09, 364.04)	337.35 (285.34, 429.74)	0.007
Fat (gr/day)	92.75 (74.04, 117.28)	104.23 (82.98, 123.77)	0.011
PRAL	-9.08 (-20.78, 3.13)	-7.71 (-21.53, 1.03)	0.827
NEAP	38.93 (32.23, 49.68)	41.52 (33.28, 50.93)	0.483
Protein/potassium	0.023 (0.020, 0.028)	0.024 (0.020, 0.029)	0.483

[1] Using Mann–Whitney U or Independent Samples T-test, as appropriate

[2] Values are median (Q1–Q3) unless otherwise noted

BMI: body mass index, PRAL: Potential Renal Acid Load, NEAP: Net Endogenous Acid Production
